# Metabolomic Profiles of Body Mass Index in the Framingham Heart Study Reveal Distinct Cardiometabolic Phenotypes

**DOI:** 10.1371/journal.pone.0148361

**Published:** 2016-02-10

**Authors:** Jennifer E. Ho, Martin G. Larson, Anahita Ghorbani, Susan Cheng, Ming-Huei Chen, Michelle Keyes, Eugene P. Rhee, Clary B. Clish, Ramachandran S. Vasan, Robert E. Gerszten, Thomas J. Wang

**Affiliations:** 1 Framingham Heart Study of the National Heart, Lung, and Blood Institute and Boston University School of Medicine, Framingham, Massachusetts, United States of America; 2 Cardiovascular Research Center, Department of Medicine, Massachusetts General Hospital, Harvard Medical School, Boston, Massachusetts, United States of America; 3 Division of Cardiology, Department of Medicine, Massachusetts General Hospital, Harvard Medical School, Boston, Massachusetts, United States of America; 4 Department of Mathematics and Statistics, Boston University, Boston, Massachusetts, United States of America; 5 Mount Auburn Hospital, Cambridge, Massachusetts, United States of America; 6 Division of Cardiology, Brigham and Women’s Hospital, Harvard Medical School, Boston, Massachusetts, United States of America; 7 Renal Division, Department of Medicine, Massachusetts General Hospital, Harvard Medical School, Boston, Massachusetts, United States of America; 8 Broad Institute of MIT and Harvard, Cambridge, Massachusetts, United States of America; 9 Division of Cardiology and Preventive Medicine, Department of Medicine, Boston University, Boston, Massachusetts, United States of America; 10 Division of Cardiovascular Medicine, Department of Medicine, Vanderbilt University, Nashville, Tennessee, United States of America; McMaster University, CANADA

## Abstract

**Background:**

Although obesity and cardiometabolic traits commonly overlap, underlying pathways remain incompletely defined. The association of metabolite profiles across multiple cardiometabolic traits may lend insights into the interaction of obesity and metabolic health. We sought to investigate metabolic signatures of obesity and related cardiometabolic traits in the community using broad-based metabolomic profiling.

**Methods and Results:**

We evaluated the association of 217 assayed metabolites and cross-sectional as well as longitudinal changes in cardiometabolic traits among 2,383 Framingham Offspring cohort participants. Body mass index (BMI) was associated with 69 of 217 metabolites (P<0.00023 for all), including aromatic (tyrosine, phenylalanine) and branched chain amino acids (valine, isoleucine, leucine). Additional metabolic pathways associated with BMI included the citric acid cycle (isocitrate, alpha-ketoglutarate, aconitate), the tryptophan pathway (kynurenine, kynurenic acid), and the urea cycle. There was considerable overlap in metabolite profiles between BMI, abdominal adiposity, insulin resistance [IR] and dyslipidemia, modest overlap of metabolite profiles between BMI and hyperglycemia, and little overlap with fasting glucose or elevated blood pressure. Metabolite profiles were associated with longitudinal changes in fasting glucose, but the involved metabolites (ornithine, 5-HIAA, aminoadipic acid, isoleucine, cotinine) were distinct from those associated with baseline glucose or other traits. Obesity status appeared to “modify” the association of 9 metabolites with IR. For example, bile acid metabolites were strongly associated with IR among obese but not lean individuals, whereas isoleucine had a stronger association with IR in lean individuals.

**Conclusions:**

In this large-scale metabolite profiling study, body mass index was associated with a broad range of metabolic alterations. Metabolite profiling highlighted considerable overlap with abdominal adiposity, insulin resistance, and dyslipidemia, but not with fasting glucose or blood pressure traits.

## Introduction

Currently one quarter of adults in the United States are obese, and it is predicted that more than half of the population will be obese by 2030 [1]. With the growing obesity epidemic, the incidence of diabetes mellitus has doubled over the last 30 years [2]. The increased risk of diabetes is most pronounced in those with a high body mass index (BMI) [2], underscoring the close link between obesity and metabolic disease. Recent advances in high-throughput technology have allowed for the systematic assessment of metabolic profiles, and have provided insights into metabolic pathways that appear dysregulated in cardiometabolic disease [3–5]. Prior studies have focused on cross-sectional associations of metabolite profiles and metabolic traits [3–5], with some recent studies relating selected metabolites to the development of future clinical disease [6–8].

Although obesity and metabolic disease traits commonly occur together, it has been increasingly recognized that a subset of obese individuals can be classified as 'metabolically healthy,’ and, in turn, lean individuals can be 'metabolically unhealthy'. This observation motivates the use of tools such as metabolomic profiling to provide a better understanding of the heterogeneity in metabolic risk among obese and lean individuals. Furthermore, we hypothesized that metabolite profiles could presage changes in metabolic traits over time.

The Framingham Heart study provides a unique community-based setting in which cardiometabolic traits have been systematically and longitudinally assessed in individuals across the spectrum of cardiometabolic risk. We investigated the comprehensive profile of non-lipid and lipid metabolites in a sample of over 2,300 community-based adults, in order to elucidate metabolic signatures of obesity and related cardiometabolic traits. We sought to determine metabolite signatures associated with cross-sectional cardiometabolic traits, as well as with their longitudinal trajectories. Using genetic markers, we explored the directionality of association between metabolites and BMI.

## Materials and Methods

### Study Sample

Participants of the Framingham Heart Study Offspring Study—a prospective, observational, community-based cohort—were examined [9]. Of 3,799 participants who attended the baseline examination cycle (1991–1995), profiling of polar positive-charge metabolites was performed on 2,526 participants with available blood samples, of whom 2,383 attended at least one subsequent examination among exam 6 (1995–1998), 7 (1998–2001), and 8 (2005–2008). Of these participants, 1,969 also underwent profiling of polar negative-charge metabolites, and 1,962 underwent lipid profiling.

Blood pressure was defined as the average of two seated resting measurements performed by a physician. BMI was calculated as weight divided by height^2^ (kg/m^2^), and waist circumference was measured at the level of the umbilicus at mid-respiration. Diabetes mellitus was defined as a fasting glucose ≥126 mg/dL, non-fasting blood glucose ≥200 mg/dL, or the use of antidiabetic medications. The homeostatic model assessment of insulin resistance was calculated as HOMA-IR [= fasting insulin (ulU/ml) x fasting glucose (mmol/ml) / 22.5] [10], and log-transformed due to a skewed distribution. Insulin resistance was estimated as outlined previously [11], using the top quartile of HOMA-IR from the whole FHS Offspring sample free of diabetes at baseline. Current smoking was defined as smoking regularly during the year prior to the examination. Total cholesterol, high-density lipoprotein (HDL) cholesterol, and triglycerides were measured after an overnight fast. All participants provided written informed consent and the study protocol was approved by the Boston University Medical Center Institutional Review Board.

### Metabolite profiling

Baseline plasma samples were collected in EDTA after an overnight fast, immediately processed, and stored at -80°C until assayed. Targeted metabolite profiling was performed using liquid chromatography with tandem mass spectrometry. Positively charged polar metabolites (including amino acids and derivatives, urea cycle intermediates, and nucleotides), lipid metabolites (lysophosphatidylcholines, LPCs; lysophosphatidylethanolamines, LPEs; phosphatidylcholines, PCs; sphingomyelins, SMs; cholesterol esters, CEs; diacylglycerols, DAGs; and triacylglycerols, TAGs), and negatively charged polar metabolites (organic acids, sugars, and bile acids) were profiled as previously described [12,13]. Nomenclature for lipid analytes entailed an initial number, denoting the total number of carbons in the lipid acyl chain, and a second number following the colon, denoting the total number of double bonds. Details are provided in S1 File and a list of 217 metabolites measured using this platform, and descriptions are detailed in **Table A in** S1 File.

### Statistical Analysis

Baseline characteristics were summarized for the sample, and correlations of metabolic traits (BMI, waist circumference, HOMA-IR, HDL cholesterol, log-triglycerides, fasting glucose, systolic and diastolic blood pressure, pulse pressure) at baseline and 3 subsequent examinations were evaluated. Metabolite concentrations were log-transformed for analysis due to right-skewed distributions.

Our primary analysis focused on metabolite associations with BMI; secondary analyses examined related metabolic traits (waist circumference, HOMA-IR, HDL, log-triglycerides, fasting glucose, systolic and diastolic blood pressure). For analyses examining HOMA-IR and fasting glucose, participants taking antidiabetic medications were excluded. Cross-sectional data for metabolic traits (response variables) and metabolites (predictors) were assessed using linear regression, adjusted for age, sex, BMI, log-triglycerides, and a multi-level indicator variable for batch number. Associations between baseline metabolites and longitudinal changes in metabolic traits were assessed using PROC GLIMMIX to account for repeated measures in a random effects model, with a class variable to indicate subsequent exam cycles. Models were adjusted for baseline metabolic trait, age, sex, BMI, log-triglycerides, and batch. In order to account for multiple testing, a P-value threshold of 0.05 / (number of metabolites tested) = 0.05 / 217 = 0.00023 was deemed statistically significant.

In exploratory analyses, we examined the association of metabolites with HOMA-IR in non-diabetic individuals stratified by obesity status. Analyses were performed using SAS, version 9.2 (SAS Institute, Cary, NC).

## Results

A total of 2,383 participants (mean age 55 ± 10 years, 53% women) underwent profiling of 217 metabolites. The mean BMI was 27.5 ± 4.9 kg/m^2^, with median BMI of 26.9 kg/m^2^ (25^th^, 75^th^ percentile 24.0, 30.0), and 25% were obese. The range of BMI is presented in S1 Fig Baseline median HOMA-IR was 2.7 mg*microU/dL*mL (25^th^, 75^th^ percentile 0.9, 2.9), and 28% were insulin resistant, defined as the upper quartile among non-diabetic individuals (Table 1).

**Table 1:** Baseline characteristics of sample.

	Total sample (n = 2383)
**Clinical characteristics**	
Age, years	55 (10)
Women, n (%)	1264 (53)
Body-mass index, kg/m^2^	27.5 (4.9)
Systolic blood pressure, mmHg	126 (19)
Diastolic blood pressure, mmHg	75 (10)
Heart rate, bpm	65 (11)
Hypertension treatment, n (%)	453 (19)
Diabetes mellitus, n (%)	154 (6)
Diabetes treatment, n (%)	69 (3)
Smoking, n (%)	435 (18)
**Laboratory values, median (25**^**th**^, **75**^**th**^ **percentile)**	
Total cholesterol, mg/dl	204 (180, 228)
High density lipoprotein cholesterol, mg/dl	47 (39, 58)
Triglycerides, mg/dl	120 (85, 178)
Fasting glucose, mg/dl	95 (89, 103)
HOMA-IR, mg*microU/dL*mL	1.7 (0.9, 2.9)
Estimated glomerular filtration rate, mL/min/1.73m^2^	90 (76, 101)

Data are presented as mean (SD), number (%), or median (25^th^, 75^th^ percentile)

HOMA-IR, homeostatic model assessment of insulin resistance

### Body mass index (BMI) is associated with broad alterations in multiple biochemical pathways

BMI was significantly associated with 69 of 217 metabolites using a conservative Bonferroni-corrected threshold (P<0.00023, Tables 2 and 3). An additional 11 metabolites displayed “suggestive” associations (P<0.001, full results in S1 Table. The top metabolite groups associated with BMI included aromatic amino acids (tyrosine, P = 3.2x10^-34^; phenylalanine P = 5.7x10^-25^), branched chain amino acids (valine, P = 2.6x10^-32^; isoleucine, P = 2.9x10^-25^; leucine, P = 2.8x10^-22^), and other amino acids (alanine, P = 1.3x10^-11^; glycine, P = 4.2x10^-10^; proline, P = 1.7x10^-4^), all of which were positively associated with BMI.

**Table 2:** Associations of non-lipid metabolite profiles with BMI and other metabolic traits.

	Body mass index		Waist circumference		Fasting glucose		HOMA-IR		HDL cholesterol		Triglycerides		Systolic BP		Diastolic BP	
Metabolites	beta	P-value	beta	P-value	beta	P-value	beta	P-value	beta	P-value	beta	P-value	beta	P-value	beta	P-value
tyrosine	1.22	3.2E-34	3.09	5.2E-32	0.21	0.64	0.07	1.0E-04	0.75	4.3E-03	0.08	3.3E-12	1.50	1.0E-04	0.60	6.0E-03
valine	1.25	2.6E-32	3.14	1.3E-29	1.52	1.6E-03	0.10	2.0E-08	-1.65	2.3E-09	0.14	2.5E-30	-0.40	0.33	-0.39	0.09
isoleucine	1.19	2.9E-25	3.06	1.6E-24	1.06	0.04	0.10	1.1E-06	-2.23	4.2E-14	0.18	4.0E-45	0.55	0.21	-0.02	0.95
phenylalanine	1.05	5.7E-25	2.76	2.4E-25	-1.07	0.02	0.05	3.7E-03	-0.98	1.9E-04	0.03	0.01	0.03	0.94	0.03	0.89
leucine	1.13	2.8E-22	3.07	9.1E-24	1.43	6.3E-03	0.08	1.2E-04	-2.01	2.4E-11	0.16	8.8E-35	-0.25	0.58	-0.25	0.32
isocitrate	1.04	8.8E-20	3.04	1.8E-24	1.97	1.0E-04	0.14	2.1E-12	0.62	0.03	0.11	3.8E-16	1.56	3.3E-04	1.03	2.5E-05
kynurenine	0.98	3.3E-19	2.64	2.4E-20	-1.62	7.8E-04	0.07	2.9E-04	-0.68	0.02	0.06	7.5E-06	0.14	0.73	0.58	0.01
2-aminoadipic acid	1.03	2.0E-18	2.53	2.4E-16	0.55	0.28	0.16	1.3E-15	-1.76	4.6E-09	0.13	7.1E-22	-0.12	0.78	0.26	0.30
fructose; glucose; galactose	0.96	5.5E-18	2.60	2.6E-19	13.27	1.3E-167	0.20	3.7E-23	-0.59	0.04	0.08	9.0E-10	1.11	8.21E-03	0.03	0.89
quinolinic acid	0.90	2.1E-16	2.14	5.3E-14	-1.77	1.8E-04	0.09	1.6E-06	-1.18	1.9E-05	0.06	1.3E-06	-0.51	0.22	-0.07	0.76
lactate	0.86	4.7E-14	2.13	5.8E-13	2.62	9.6E-08	0.17	4.6E-20	0.68	0.02	0.18	1.2E-45	2.24	1.3E-07	1.06	9.9E-06
phosphocreatine	-0.80	6.3E-13	-1.99	7.1E-12	-1.49	1.7E-03	-0.06	1.2E-03	0.28	0.32	-0.02	0.07	-0.50	0.23	0.26	0.27
alanine	0.69	1.3E-11	1.87	2.5E-12	1.17	8.7E-03	0.09	8.6E-08	0.15	0.57	0.17	4.8E-54	1.27	9.0E-04	0.75	5.8E-04
glycerol	0.79	2.1E-11	1.93	3.9E-10	0.97	0.03	0.03	0.08	2.64	1.4E-18	0.14	3.3E-25	1.88	1.5E-05	0.69	5.2E-03
kynurenic acid	0.65	2.9E-11	1.62	2.4E-10	-0.15	0.72	0.04	0.03	0.08	0.73	-0.02	0.04	0.68	0.06	0.35	0.09
alpha-ketoglutarate	0.75	6.3E-11	1.94	6.9E-11	1.26	9.8E-03	0.09	9.9E-07	0.47	0.10	0.08	5.3E-10	0.51	0.23	0.47	0.05
glycine	-0.62	4.2E-10	-1.52	4.5E-09	-1.66	1.2E-04	-0.07	7.2E-05	-1.08	1.7E-05	-0.09	1.0E-14	-0.95	0.01	-0.44	0.04
alpha-hydroxybutyric acid	0.67	5.1E-10	1.73	7.6E-10	4.10	1.2E-18	0.05	4.0E-03	1.62	2.1E-09	0.09	1.5E-12	1.19	3.2E-03	0.54	0.02
choline	0.58	1.4E-08	1.54	9.9E-09	-0.88	0.05	-0.04	0.01	0.46	0.07	0.06	1.9E-06	0.17	0.66	0.12	0.57
carnitine	0.52	1.6E-07	1.39	1.2E-07	-1.11	0.01	-0.01	0.53	-0.65	0.01	0.05	6.2E-05	-0.85	0.02	-0.61	3.8E-03
glycero-phosphocholine	-0.48	3.6E-07	-1.01	4.4E-05	-1.50	2.6E-04	0.02	0.29	0.78	1.1E-03	0.05	9.5E-06	-0.14	0.70	0.17	0.39
xanthosine	0.51	3.8E-07	0.95	2.9E-04	-1.02	0.02	0.04	8.7E-03	-0.47	0.06	0.06	4.2E-08	0.39	0.29	0.15	0.47
carbamoylalanine	0.49	5.2E-07	1.26	9.5E-07	0.64	0.13	0.10	2.5E-09	0.12	0.63	0.04	1.1E-04	0.73	0.05	0.59	4.1E-03
uric acid	0.51	3.3E-06	1.50	1.4E-07	-0.68	0.15	0.03	0.12	0.62	0.02	0.03	7.6E-03	-0.09	0.83	0.16	0.50
creatine	0.49	4.6E-06	1.35	1.1E-06	0.40	0.38	0.03	0.06	0.46	0.09	0.04	1.4E-03	-0.44	0.27	-0.26	0.25
aconitate	0.51	5.2E-06	1.34	4.2E-06	1.33	5.5E-03	0.07	1.1E-04	0.35	0.21	0.06	6.3E-07	1.31	1.5E-03	0.96	4.0E-05
cotinine	-0.40	2.1E-05	-0.35	0.15	-0.21	0.61	-0.02	0.13	-1.15	1.6E-06	0.04	1.2E-03	-0.44	0.21	-0.65	1.1E-03
ornithine	0.39	7.2E-05	0.97	1.5E-04	-0.86	0.04	0.03	0.07	-1.23	5.3E-07	0.01	0.27	-0.72	0.05	-0.22	0.29
citrulline	-0.39	8.6E-05	-0.87	8.0E-04	-0.92	0.03	-0.04	7.4E-03	-0.03	0.92	-0.04	3.9E-04	-0.80	0.03	-0.28	0.18
dimethylglycine	0.39	1.2E-04	1.04	7.9E-05	-1.33	2.4E-03	0.05	7.0E-03	-1.46	7.9E-09	-0.01	0.43	-0.69	0.07	-0.51	0.02
proline	0.38	1.7E-04	1.00	1.6E-04	0.21	0.64	0.07	1.8E-05	-1.00	7.7E-05	0.09	1.4E-16	1.00	7.5E-03	0.27	0.20

Analyses adjusted for age, sex, batch, body mass index (except for body mass index and waist circumference), and log-triglyceride concentrations (except for triglyceride analyses). Beta estimates indicate change in metabolic trait per 1-standard deviation increase in log-transformed metabolite.

**Table 3:** Associations of lipid metabolite profiles with BMI and other metabolic traits.

	Body mass index		Waist circumference		Fasting glucose		HOMA-IR		HDL cholesterol		Triglycerides		Systolic BP		Diastolic BP	
Metabolites	beta	P-value	beta	P-value	beta	P-value	beta	P-value	beta	P-value	beta	P-value	beta	P-value	beta	P-value
LPC 18:2	-1.15	3.2E-25	-3.03	5.8E-26	-0.23	0.64	-0.08	5.2E-05	2.03	8.4E-13	-0.04	7.3E-03	-0.81	0.06	-0.26	0.28
LPC 18:1	-1.03	7.2E-22	-2.43	3.5E-18	-1.76	1.9E-04	-0.08	7.6E-06	1.60	4.8E-09	0.07	6.0E-09	-0.65	0.11	-0.13	0.56
LPE 16:0	-1.01	2.6E-18	-2.42	1.1E-15	-0.83	0.10	-0.08	2.8E-05	2.85	1.3E-22	0.25	2.7E-103	0.78	0.08	0.41	0.10
PCA 36:4	-0.87	2.4E-16	-2.24	4.7E-16	-0.14	0.76	-0.06	1.2E-03	3.92	1.2E-50	0.03	9.6E-03	-0.01	0.97	0.06	0.77
LPC 22:6	-0.84	3.0E-15	-2.16	5.6E-15	-0.59	0.20	-0.05	4.5E-03	1.53	1.2E-08	0.06	8.6E-07	-0.06	0.87	0.14	0.53
TAG 50:1	1.09	7.8E-14	3.16	4.8E-16	1.33	0.06	0.13	1.3E-06	2.06	1.2E-07	0.36	4.3E-180	2.86	6.6E-07	1.04	1.3E-03
TAG 52:1	1.29	3.4E-13	3.84	9.1E-17	1.21	0.12	0.18	4.0E-09	1.44	1.3E-03	0.45	<1E-300	2.50	1.8E-04	1.03	6.3E-03
LPE 18:2	-0.73	5.8E-12	-1.84	2.1E-11	-0.09	0.84	-0.06	6.5E-04	2.11	1.4E-15	0.10	2.3E-15	-0.02	0.95	-0.13	0.56
LPE 18:1	-0.72	8.4E-12	-1.86	1.5E-11	-1.26	6.5E-03	-0.05	0.01	2.09	3.4E-15	0.12	1.6E-22	0.06	0.88	0.17	0.45
LPC 20:4	-0.73	1.2E-11	-1.87	2.7E-11	-0.29	0.54	-0.07	2.7E-04	2.65	5.8E-23	0.05	3.8E-05	-0.05	0.91	0.06	0.79
SM 18:1	0.74	1.4E-11	1.52	1.1E-07	-0.28	0.56	0.00	1.00	-0.22	0.42	-0.06	2.8E-06	-1.10	7.5E-03	-0.26	0.27
SM 18:0	0.71	4.2E-11	1.68	1.7E-09	0.95	0.04	0.05	6.1E-03	0.05	0.86	-0.03	0.03	-0.60	0.13	-0.13	0.56
TAG 54:4	-0.73	5.7E-11	-2.04	1.6E-12	-1.23	9.7E-03	-0.04	0.04	-3.57	6.4E-39	0.19	2.5E-60	-2.57	4.5E-10	-1.09	3.1E-06
LPE 18:0	-0.74	2.3E-10	-1.72	1.5E-08	-1.21	0.02	-0.06	4.0E-03	2.16	9.4E-14	0.23	4.0E-89	-0.15	0.73	0.31	0.21
LPE 20:4	-0.69	2.5E-10	-1.71	1.5E-09	0.59	0.21	-0.09	1.2E-06	3.25	7.2E-34	0.14	4.0E-29	0.76	0.06	0.30	0.19
TAG 50:2	0.95	2.9E-10	2.69	6.9E-12	-0.51	0.43	0.06	0.01	2.64	3.0E-12	0.39	1.8E-280	2.92	2.1E-07	1.16	2.6E-04
TAG 48:0	0.75	2.6E-09	2.35	5.3E-13	0.36	0.51	0.12	1.7E-08	2.14	8.6E-12	0.28	2.9E-127	2.90	4.6E-10	1.19	5.4E-06
TAG 48:1	0.79	5.5E-09	2.39	1.5E-11	0.15	0.80	0.11	3.5E-06	2.76	4.6E-16	0.34	3.9E-197	3.29	7.7E-11	1.42	6.7E-07
SM 16:1	0.63	2.8E-08	1.48	6.6E-07	-0.07	0.89	-0.05	8.9E-03	2.83	1.8E-23	-0.01	0.53	-0.32	0.45	0.14	0.56
TAG 56:3	-0.88	4.2E-08	-2.10	4.6E-07	-0.33	0.63	-0.02	0.35	-1.17	3.3E-03	0.41	<1E-300	-1.83	2.1E-03	-0.96	4.1E-03
TAG 56:4	-0.74	5.6E-08	-1.57	9.2E-06	-0.04	0.94	-0.05	0.02	-0.94	5.7E-03	0.34	7.8E-218	-1.44	4.1E-03	-0.79	5.2E-03
TAG 46:0	0.66	8.9E-07	2.11	4.7E-09	0.07	0.91	0.12	8.4E-07	2.38	2.3E-11	0.30	3.6E-133	2.89	3.4E-08	1.20	4.3E-05
CE 20:3	0.58	1.1E-06	1.76	1.4E-08	-1.39	6.7E-03	0.02	0.29	1.23	3.8E-05	-0.26	3.9E-118	1.29	3.6E-03	0.57	0.02
PC 38:3	0.55	3.0E-06	1.61	1.2E-07	-0.15	0.77	-0.01	0.59	2.08	7.6E-13	0.20	8.3E-58	1.55	3.4E-04	0.92	1.7E-04
TAG 56:9	-0.59	5.2E-06	-1.23	2.8E-04	0.02	0.97	0.00	0.87	0.26	0.42	0.31	3.3E-173	-0.25	0.60	-0.35	0.19
TAG 54:5	-0.55	5.9E-06	-1.42	7.6E-06	-0.63	0.22	-0.03	0.10	-2.99	2.6E-23	0.27	1.7E-129	-2.10	2.9E-06	-0.81	1.4E-03
LPC 16:1	-0.51	7.2E-06	-0.91	2.2E-03	-1.12	0.02	-0.07	2.8E-04	2.09	1.2E-13	0.19	5.0E-59	0.47	0.27	0.35	0.14
PC 38:6	-0.48	8.5E-06	-1.20	2.2E-05	0.52	0.26	-0.05	9.5E-03	2.45	6.3E-20	0.10	7.1E-17	0.02	0.96	0.11	0.62
LPC 16:0	-0.50	1.2E-05	-0.80	7.0E-03	-0.91	0.06	-0.03	0.14	2.06	2.4E-13	0.18	5.2E-47	0.35	0.41	0.35	0.15
TAG 46:1	0.63	1.6E-05	1.91	5.3E-07	0.18	0.77	0.13	1.4E-07	2.60	6.8E-13	0.37	1.2E-255	3.31	8.0E-10	1.38	6.0E-06
PC 38:5	-0.46	4.3E-05	-0.95	1.3E-03	-0.40	0.41	-0.08	3.5E-05	3.29	1.8E-32	0.17	1.7E-41	-0.02	0.96	0.05	0.84
LPE 22:6	-0.44	6.4E-05	-0.81	4.6E-03	-1.51	1.4E-03	0.00	0.84	1.12	4.4E-05	0.14	2.8E-33	-0.35	0.39	0.11	0.64
PC 36:2	-0.45	6.6E-05	-1.13	1.1E-04	0.30	0.52	-0.01	0.68	2.56	2.4E-20	0.16	4.5E-39	-0.32	0.44	-0.05	0.81
LPC 20:5	-0.42	8.6E-05	-0.83	3.3E-03	-0.47	0.31	-0.05	3.7E-03	2.81	4.1E-26	0.05	4.4E-05	0.71	0.08	0.49	0.03
TAG 52:2	0.68	8.8E-05	1.81	5.5E-05	-0.85	0.24	0.03	0.26	-2.01	2.8E-06	0.43	<1E-300	-0.03	0.97	-0.58	0.11
TAG 48:2	0.65	1.3E-04	2.12	1.6E-06	-0.68	0.35	0.11	1.4E-04	2.59	8.4E-10	0.43	<1E-300	3.47	2.7E-08	1.55	1.1E-05
LPC 18:0	-0.42	1.6E-04	-0.77	7.3E-03	-1.30	5.9E-03	0.00	0.91	1.01	2.4E-04	0.14	1.9E-30	-0.26	0.53	0.08	0.73
TAG 56:10	-0.47	1.8E-04	-1.84	1.4E-08	-1.23	0.02	-0.03	0.16	-0.85	6.0E-03	0.29	1.2E-151	0.11	0.81	0.15	0.58

LPC, lysophosphatidylcholine; LPE, lysophosphatidylethanolamine; PC, phosphatidylcholine; SM, sphingomyelins; CE, cholesterol esters; DAG, diacylglycerol; TAG, triacylglycerolAnalyses adjusted for age, sex, batch, body mass index (except for body mass index and waist circumference), and log-triglyceride concentrations (except for triglyceride analyses). Beta estimates indicate change in metabolic trait per 1-standard deviation increase in log-transformed metabolite.

In addition, BMI was positively associated with multiple metabolites in the citric acid cycle (isocitrate, P = 8.8x10^-20^; alpha-ketoglutarate, P = 6.3x10^-11^; aconitate, P = 5.2x10^-6^) and the tryptophan pathway (kynurenine, P = 3.3x10^-19^; kynurenic acid, P = 2.9x10^-11^). Other pathways associated with BMI included the urea cycle (citrulline, P = 8.6x10-5; ornithine, P = 7.2x10^-5^), nucleic acid metabolism (xanthosine, P = 3.8x10^-7^; uric acid, P = 3.3x10^-6^), and multiple creatine-related metabolites (carnitine, P = 1.6x10^-7^; choline, P = 1.4x10^-8^; glycero-phosphocholine, P = 3.6x10^-7^).

### Lipid subspecies reveal a distinct pattern in obesity

**Fig 1:**
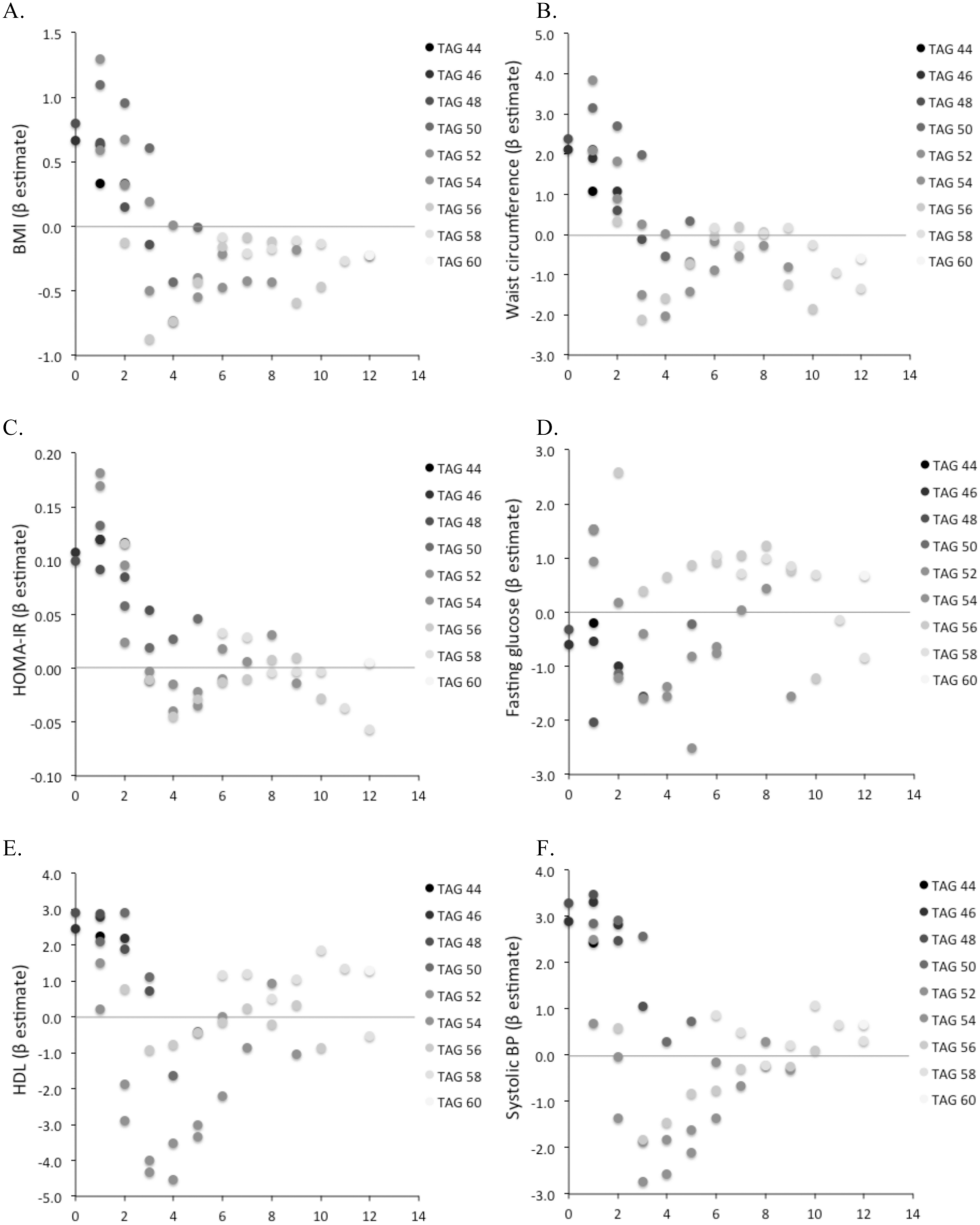
Association of metabolic traits and TAG length and saturation. X-axis represents number of double bonds, Y-axis is the beta-coefficient for the change in metabolic trait per 1 standard deviation increase in metabolite. Darker circles are shorter length TAGs, and lighter circles represent greater carbon length TAGs. Panel (A) shows body-mass index, (B) waist circumference, (C) HOMA-IR, (D) fasting glucose, (E) HDL cholesterol, and (F) systolic blood pressure.

Of 104 lipid subspecies profiled, 38 were significantly associated with BMI, including glycerol, an integral component of TAGs (P = 2.1x10^-11^), choline, and glycero-phosphocholine, both important components of PCs and SMs (P = 1.4x10^-8^; P = 3.6x10^-7^, respectively). In addition, BMI was associated with LPCs, LPEs (inversely associated with BMI), SMs, CEs (positively associated with BMI), PCs and TAGs (both positive and negative associations, P<0.00023 for all). A distinct pattern emerged within TAG subspecies; shorter carbon length and a lower number of double bonds were associated with higher BMI, whereas longer carbon length and higher number of double bonds were associated with a lower BMI (Fig 1A). The converse was observed with LPEs, where shorter carbon length and lower number of double bonds were associated with lower BMI (full results in S1 Table). No such pattern was discernible for PCs or LPCs.

### Metabolite profiles of obesity are linked to insulin resistance and dyslipidemia

**Fig 2:**
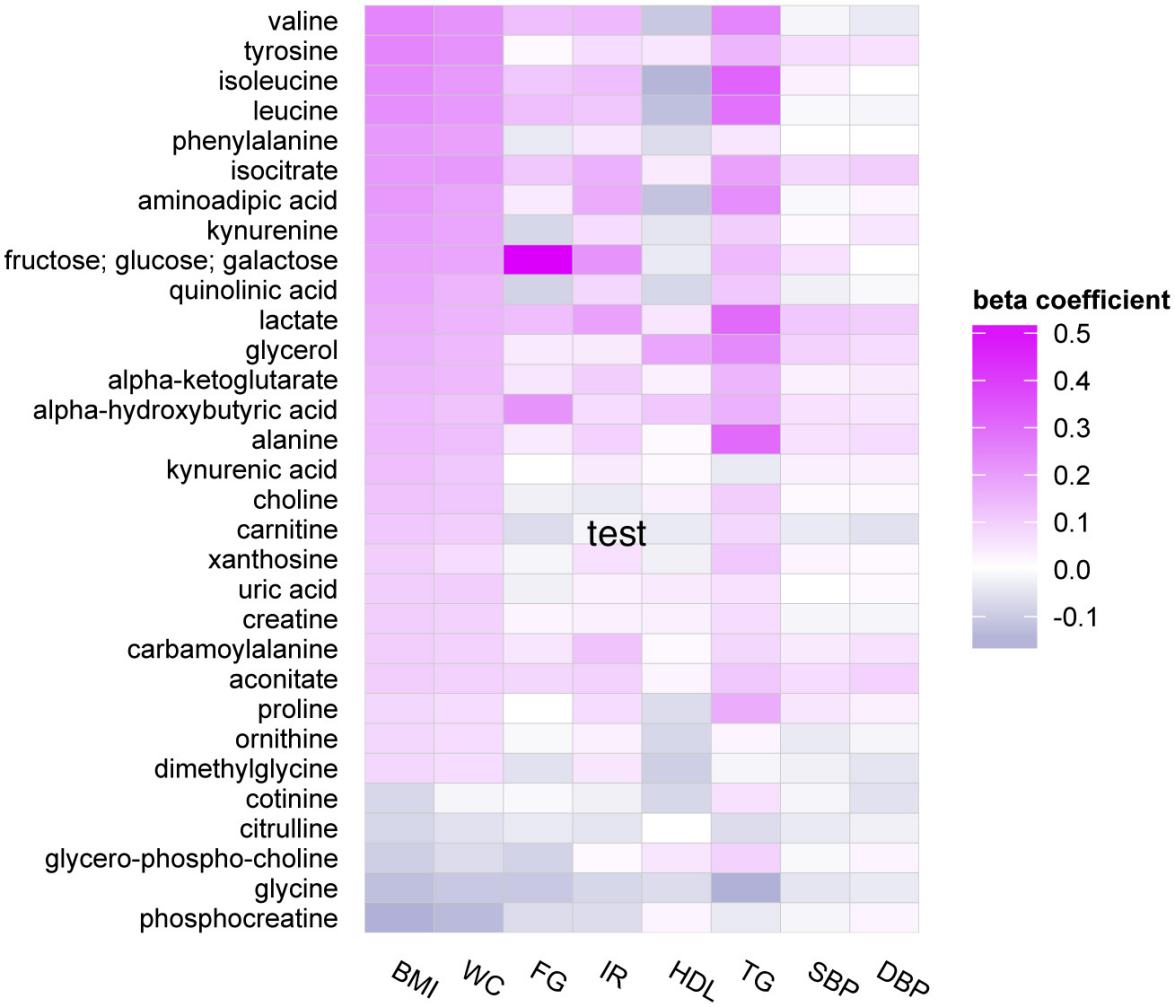
Heatmap of non-lipid metabolite profiles associated with metabolic traits.

**Fig 3:**
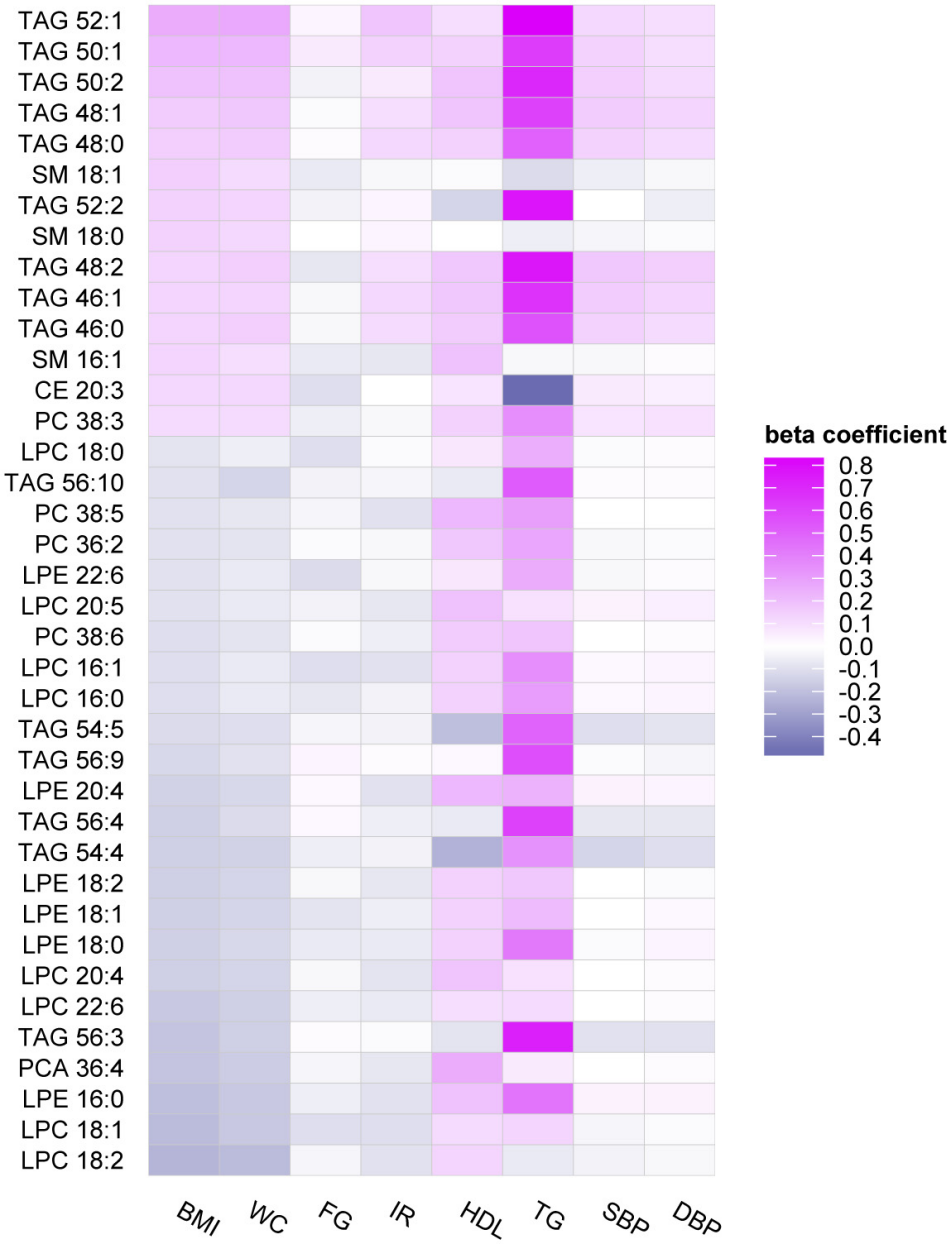
Heatmap of lipid metabolite profiles associated with metabolic traits.

The majority of metabolites associated with BMI were concomitantly associated with abdominal adiposity, as assessed by waist circumference. There was also overlap in metabolic profiles associated with BMI and other metabolic traits, including insulin resistance as estimated by HOMA-IR, HDL cholesterol, log-triglyceride concentrations, and fasting glucose, as displayed in the heatmap (Figs 2 and 3, Tables 2 and 3). Of 31 non-lipid metabolites associated with BMI, 27 demonstrated at least suggestive associations (at P<0.001) with at least one other metabolic trait other than waist circumference, after adjusting for BMI (HOMA-IR, n = 16; HDL, n = 13; log-triglycerides, n = 23; fasting glucose, n = 8). Of these, 12 metabolites were associated with at least 4 metabolic traits: isoleucine, leucine, proline, valine, and aminoadipic acid were associated with BMI, HOMA-I77R, HDL, and triglycerides; alpha-hydroxybutyric acid was associated with BMI, fasting glucose, HDL, and triglycerides; fructose/glucose/galactose, kynurine, isocitrate, and lactate were associated with BMI, fasting glucose, HOMA-IR, and triglycerides; glycine, and quinolinic acid were associated with all 5 metabolic traits. By contrast, there was limited overlap of metabolites associated with BMI and blood pressure traits, with only 5 of the 31 non-lipid BMI metabolites found in association with either systolic and/or diastolic blood pressure.

TAG patterns associated with BMI were similar for waist circumference and HOMA-IR, with shorter carbon length and lower number of double bonds (e.g. more saturation) associated with worse metabolic profiles. By contrast, there was no distinct association between TAG characteristics and fasting glucose. Lastly, TAG patterns observed in association with HDL and blood pressure traits appeared J-shaped in distribution—with low carbon length, saturated TAGs associated with higher HDL and blood pressure on the one hand, and intermediate length and saturation TAGs associated with lower HDL and lower blood pressure on the other hand (Fig 1). Full results of metabolic traits and metabolic profile analyses are shown in S1 Table.

### Baseline metabolite profiles associated with longitudinal changes in metabolic traits are distinct from cross-sectional findings

The correlation of baseline and longitudinal metabolic traits over approximately 14 years is presented in **Table B in** S1 File. There was a strong correlation of baseline BMI, waist circumference, and HDL cholesterol with subsequent measures (Pearson correlation coefficient r > 0.70 for all), but lower correlation of baseline fasting glucose, triglycerides, systolic and diastolic blood pressure with follow-up measures (r = 0.36–0.56 for baseline versus last exam).

Metabolites associated with longitudinal changes in fasting glucose were distinct from metabolites associated with fasting glucose cross-sectionally. Specifically, isoleucine (P = 1.1x10^-4^), ornithine (P = 2.2x10^-5^), 5-HIAA (P = 1.1x10^-4^), aminoadipic acid (P = 5.8x10^-5^), and cotinine (P = 3.5x10^-5^), all predicted future changes in fasting glucose, despite not being associated with baseline fasting glucose values (Table 4). By contrast, there were no metabolites associated with longitudinal changes in the remaining metabolic traits (BMI, waist circumference, HDL, triglycerides, blood pressure traits) that were not already associated in cross-sectional analyses.

**Table 4:** Metabolites associated with longitudinal changes in fasting glucose.

	Longitudinal change in fasting glucose		
Metabolite	Beta	SE	P-value
fructose; glucose; galactose	2.25	0.45	5.71E-07
ornithine	1.19	0.28	2.22E-05
cotinine	1.14	0.27	3.47E-05
aminoadipic acid	1.41	0.35	5.85E-05
isoleucine	1.33	0.34	1.11E-04
5- hydroxyindoleacetic acid	-1.30	0.34	1.14E-04
leucine	1.20	0.35	6.17E-04

Beta estimates represent change in fasting glucose per year, per 1-standard deviation change in log-metabolite. Analyses adjusted for age, sex, batch, body mass index, and log-triglyceride concentrations.

### Differential effect of metabolites on insulin resistance in obese and non-obese individuals

In exploratory analyses, we compared metabolite profiles associated with insulin resistance, as estimated by HOMA-IR, separately in obese (n = 511) and non-obese (n = 1680), non-diabetic individuals. Many metabolites associated with insulin resistance appear to be consistent across BMI strata (**Tables A and B in** S1 File). However, obesity status appeared to “modify” the association for 9 of the metabolites, with suggestive P-values for interaction (P≤0.01 for all, Table 5). This included amino acids and related metabolites (3-hydroxyphenylacetic acid, tryptophan, tyrosine, isoleucine), bile acids (glycocholate, taurocholate), and others (carnitine, creatine, inosine). All metabolites appeared more strongly associated with HOMA-IR among the non-obese, with exception of isoleucine, which appeared to have a stronger association with HOMA-IR among the obese. Of note, none of the P-values for interaction met the Bonferroni-corrected P-value threshold.

**Table 5:** Association of metabolite profiles and HOMA-IR in obese and non-obese individuals.

	Obese (n = 511)			Non-obese (n = 1680)			
	Beta est	s.e.	P-value	Beta est	s.e.	P-value	P for interaction
3-hydroxyphenylacetic acid	0.01	0.02	0.52	0.12	0.03	3.47E-04	**1.40E-03**
carnitine	-0.02	0.02	0.36	0.07	0.03	0.05	**1.70E-03**
glycocholate	0.03	0.02	0.09	0.12	0.03	4.50E-04	3.89E-03
tryptophan	0.02	0.02	0.33	0.06	0.03	0.07	0.01
tyrosine	0.05	0.02	0.02	0.11	0.03	1.52E-03	0.01
creatine	0.03	0.02	0.21	0	0.04	0.96	0.01
isoleucine	0.08	0.02	8.90E-04	0.10	0.04	0.01	0.01
taurocholate	0.09	0.02	**1.45E-05**	0.18	0.03	**1.11E-08**	0.01
inosine	0.00	0.02	0.82	0.11	0.04	3.37E-03	0.01

Beta estimates represent change in HOMA-IR per 1-standard deviation change in log-metabolite. Analyses adjusted for age, sex, batch, body mass index, and log-triglyceride concentrations. Bold font indicates P-value below Bonferroni-corrected threshold of P<0.00023.

## Discussion

Our findings are threefold: first, BMI is associated with broad alterations in metabolites representing multiple distinct biochemical pathways, including the metabolism of amino acids, the citric acid cycle, nucleotides, the urea cycle, and specific triacylglycerol species. Second, there was considerable overlap in metabolite profiles associated with BMI and other metabolic traits, including abdominal adiposity, insulin resistance, and dyslipidemia. By contrast, profiles of fasting glucose and blood pressure traits were relatively distinct. Lastly, obesity appeared to modify metabolic signatures of insulin resistance. Collectively, these data highlight the important interaction of obesity and metabolic health—and reveal with further granularity the intricateness of this relationship.

A number of recent studies have identified a range of metabolites as markers and possible effectors of metabolic disease [3,4,8,14–18]. We extend these analyses to a broad range of cardiometabolic phenotypes and present a more detailed analysis of metabolite profiles with respect to adiposity, dyslipidemia, metabolic disease, and blood pressure. Our group has previously examined metabolites in relation to these traits [5], however we now extend findings to more than double the previous sample size, with a much broader profiling platform that now includes negatively charged anions and lipid subspecies. A number of novel findings are worth noting. For example, mounting evidence supports the role of dysregulated branched-chain amino acid metabolism in human cardiometabolic disease [6,7,14,16,19–21], with experimental studies demonstrating activation of the mammalian target of rapamycin (mTOR) by branched chain amino acids leading to insulin resistance [14,22,23]. Our study demonstrates not only that branched-chain and aromatic amino acids are associated with BMI, but also other essential and non-essential amino acids (alanine, asparagine, glycine, proline) and amino acid intermediates. The latter included multiple metabolites of the tryptophan pathway (indole propionate, kynurenic and quinolinic acid), cystathionine in the cysteine pathway, and 2-aminoadipic acid, a lysine metabolite, which has previously been associated with incident diabetes [24]. Prior studies have demonstrated lower glycine concentrations to be associated with metabolic traits, worse glucose disposal rate, and diabetes [14,16,25,26]. We now confirm the association of lower glycine concentrations with unfavorable metabolic traits, and moreover show that its derivative metabolite, dimethylglycine, is positively associated with metabolic traits.

We observed that most amino acids associated with BMI were also associated with abdominal adiposity, insulin-resistance, HDL, and triglycerides. However, two essential amino acids were negatively associated with fasting glucose but not with BMI: histidine, which has been shown to protect against diabetic deterioration in mice [27], and threonine, a precursor to glycine synthesis. Among non-amino acid metabolites, considerable overlap in metabolite profiles existed between BMI, abdominal adiposity, insulin resistance, and lipid traits, but not with elevated fasting glucose, highlighting two known distinct metabolic phenotypes. Whereas impaired fasting glucose reflects predominantly a defect in insulin secretion, impaired glucose tolerance is more closely related to insulin resistance [28,29] and clinical outcomes [30]. Our study provides further granularity with respect to differences in metabolite intermediaries between these two phenotypes.

Multiple additional metabolic pathways appear to be altered in relation to metabolic traits, including carbohydrate metabolism, ketosis, lipid metabolism, and bile acid metabolism [3,4,8,14,17,31–33]. Our findings affirm the effect of BMI and related traits on this wide spectrum of metabolic pathways, and extend previous findings in two ways. First, our broad-based platform was able to shed further light regarding multiple metabolic intermediates involved in related pathways. For example, we demonstrate novel alterations in multiple intermediates of the citric acid cycle, including aconitate, isocitrate, and alpha-ketoglutarate, all of which are positively associated with BMI, abdominal obesity, HOMA-IR, and triglycerides. A previous study demonstrated that alpha-ketoglutarate was reduced after weight loss in obese insulin-resistant women [34]. Further, the citric acid cycle enzyme NADP+-dependent isocitrate dehydrogenase is expressed in liver and adipocytes, where it produces NADPH by converting isocitrate to alpha-ketoglutarate, and thereby plays a critical role in adipogenesis, particularly with respect to triglycerides and cholesterol [35].

Previous studies have demonstrated an inverse association of long-chain acylcarnitines [32], positive association with medium-chain acylcarnitines [31], and also greater fatty acid saturation levels [3,4,18] with metabolic traits. We extend these findings and demonstrate a distinct TAG pattern associated with BMI, central adiposity, and HOMA-IR, with lower carbon length and saturation associated with worse metabolic profiles. This finding is in concert with prior lipidomic studies, that have demonstrated the association of low carbon number and double-bond content TAG subspecies with incident cardiovascular disease [36]. Whether TAG subspecies are markers or effectors of disease remains unclear, however it is known that triglycerides may modulate lipoprotein retention and thrombogenicity as potential underlying mechanisms of atherosclerosis [37]. Together, these findings suggest that profiling of specific TAG species beyond conventional assessment of bulk triglyceride concentrations may lend more granular insights into underlying pathways contributing to cardiometabolic disease.

Interestingly, we found no metabolites that predicted change in BMI over time above and beyond baseline BMI. This is probably due to the fact that baseline and subsequent BMI measurements are highly correlated. By contrast, we found multiple metabolites that predicted future change in fasting glucose measures, including aminoadipic acid, ornithine, isoleucine, 5-HIAA, and cotinine, each of which has support from previous studies linking metabolite to glucose homeostasis and diabetes mellitus: Our group has previously demonstrated an association of 2-aminoadipic acid with incident diabetes [24]. In this study, administration of 2-aminoadipic acid to mice lowered their fasting glucose levels. Further, in cell-based experiments, 2-aminoadipic acid enhanced insulin secretion from pancreatic beta cell lines. This suggests that 2-aminoadipic acid may be involved in glucose homeostasis and underlying pathology. Ornithine is an amino acid that is part of the urea cycle. In animal models of diabetes, insulin deficiency was associated with increased levels of ornithine along with high branched chain amino acid levels such as isoleucine, [38] and recapitulates hyperaminoacidemia seen in human metabolic disease that precedes diabetes [6]. Amino acid supplementation in humans directly promotes insulin resistance [23], and may be related to effects on insulin signaling in skeletal muscle [39]. 5-HIAA is a metabolite of serotonin, a neurotransmitter that plays a central role in glucose homeostasis via central autonomic pathways and peripheral release from gastrointestinal enterochromaffin cells [40]. Genetic serotonin receptor deletion is associated with diabetes mellitus, further substantiating the role of serotonin in diabetes development [41]. Lastly, cotinine is the predominant metabolite of nicotine, and can be used as a marker of smoking status [42], which in turn has been linked to incident diabetes in cohort studies [43]. Smoking has been shown to lead to impaired glucose tolerance acutely [44], and abdominal adiposity [45], both of which may lead to eventual diabetes mellitus.

It is well recognized that within a given BMI category, a range of metabolic health phenotypes can be observed. Whether or not 'metabolically healthy obesity' exists is controversial [46], but it is known that heterogeneity exists in the relation of metabolic profiles with metabolic health across weight categories [19]. Our findings highlight the complex interaction of obesity with metabolic health, and demonstrate notable differences in metabolic signatures of insulin resistance in obese compared with non-obese individuals. For example, the amino acids isoleucine and proline were more strongly associated with insulin resistance in the non-obese. By contrast, the association of saturated TAGs and both conjugated and deconjugated bile acids with insulin resistance was markedly more pronounced in obesity. The association of bile acids and metabolic traits has previously been described [3,47–49]. Differences in obese compared with non-obese may also relate to the rate-limiting enzyme of bile acid synthesis, cholesterol 7α-hydroxylase (CYP7A), whose basal expression is increased in an obesity mouse model via both glucose-stimulated epigenetic modifications and the insulin/FoxO1 pathway [50]. Alterations in gut microbiota may also contribute, as intestinal flora can modify bile acids by deconjugation, oxidation, dehydroxylation, and sulfation [51], and have been implicated in the pathogenesis of diabetes [52].

Several limitations deserve mention. The 217 metabolites analyzed in this study were selected as part of a targeted platform geared toward small molecules based on previous studies linking several metabolites to insulin resistance [6]. Other metabolite platforms can acquire profiles in a less biased manner, however advantages for our approach included highly specific analyte identification, and capability for absolute quantification of metabolites. It remains unclear whether metabolites represent biomarkers or actual mediators of metabolic disease, and causal inferences cannot be drawn from our observational study. There were 154 individuals with diabetes mellitus in our sample, and we thus we had limited power to examine the association of metabolites with metabolic traits among this subset. We were unable to ascertain relations of plasma metabolites to the adipose tissue secretome, as these data were not available in our study sample. Lastly, we performed metabolite profiles at one single time point, precluding the assessment of cardiometabolic disease and medication effects on longitudinal changes in metabolites.

In summary, body mass index was associated with a broad range of metabolic alterations. Metabolite profiling illuminated considerable overlap with abdominal adiposity, insulin resistance, and dyslipidemia, but not with fasting glucose or blood pressure traits. The association of metabolic profiles with IR was modified by the presence or absence of obesity, highlighting heterogeneity in the association of obesity and metabolic disease. Future studies are needed to identify which metabolites could have importance as causal risk factors for metabolic disease, and, in turn, which metabolites could serve as potential targets for therapy.

## Supporting Information

S1 Fig Distribution of baseline body mass index in the sample. (PDF)

S1 File Supplemental information to manuscript. Supplemental methods are included in text. Metabolites assayed and descriptions are summarized (Table A), and correlations of metabolic traits across subsequent examinations displayed (Table B). Results for the association of non-lipid (Table C) and lipid (Table D) with HOMA-IR in obese and non-obese individuals are summarized.
(DOCX)

S1 Table Full results of metabolite associations with cardiometabolic traits. (XLSX)
