# Supplementary figures and images for: Metabolomic Profiles of Body Mass Index in the Framingham Heart Study Reveal Distinct Cardiometabolic Phenotypes
Source: PLoS One. 2016 Feb 10;11(2):e0148361. doi: 10.1371/journal.pone.0148361 (PMC4749349; doi:10.1371/journal.pone.0148361)

SFig 1. Distribution of BMI in FHS sample

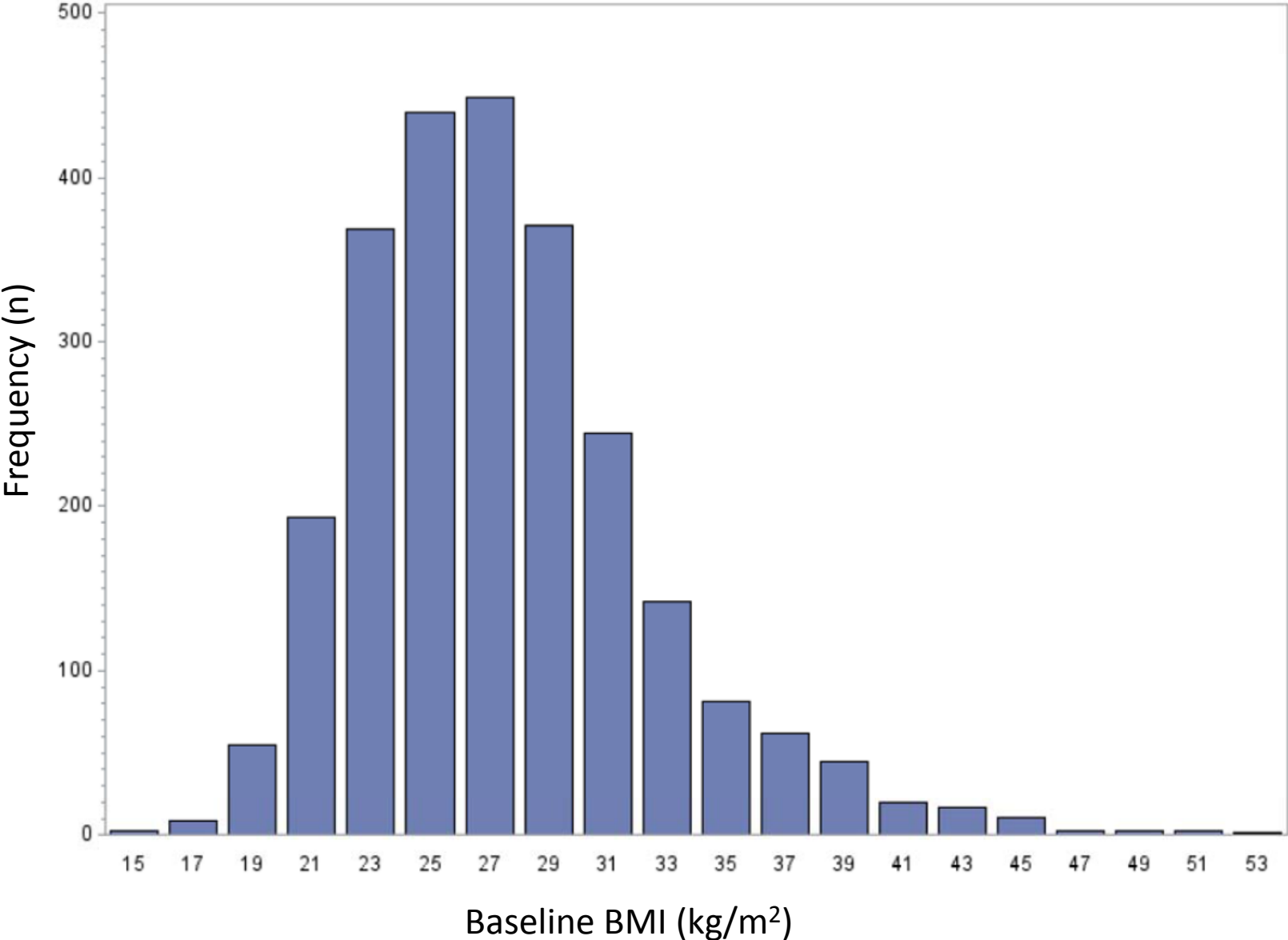

Supplement: S1 Fig — (PDF) [file pone.0148361.s001.pdf]
