# Supplementary material for: Metabolomic Profiles of Body Mass Index in the Framingham Heart Study Reveal Distinct Cardiometabolic Phenotypes
Source: PLoS One. 2016 Feb 10;11(2):e0148361. doi: 10.1371/journal.pone.0148361 (PMC4749349; doi:10.1371/journal.pone.0148361)
Supplement: S1 File — Supplemental methods are included in text. Metabolites assayed and descriptions are summarized (Table A), and correlations of metabolic traits across subsequent examinations displayed (Table B). Results for the association of non-lipid (Table C) and lipid (Table D) with HOMA-IR in obese and non-obese individuals are summarized. (DOCX) [file pone.0148361.s002.docx]

**Supporting Information (S1 File)**

**Metabolite Profiling of Obesity in the Framingham Heart Study Reveal Distinct Cardiometabolic Phenotypes**

**Contents**

**Methods. Supplemental Methods**

**Table A. Metabolites assayed and descriptions.**

**Table B. Correlation of metabolic traits across examinations**

**Table C. Association of non-lipid metabolites and HOMA-IR in obese and non-obese individuals.**

**Table D. Association of lipid metabolites and HOMA-IR in obese and non-obese individuals.**

**Methods**

**Metabolite profiling**

Baseline plasma samples were collected in EDTA after an overnight fast, immediately processed, and stored at -80°C until assayed. Targeted metabolite profiling was performed using liquid chromatography with tandem mass spectrometry. In brief, positively charged polar metabolites (including amino acids and derivatives, urea cycle intermediates, and nucleotides) were profiled beginning with 10 μL of EDTA plasma, extracted with acetonitrile and methanol and separated by hydrophilic interaction liquid chromatography [1]. Lipid metabolites (lysophosphatidylcholines, LPCs; lysophosphatidylethanolamines, LPEs; phosphatidylcholines, PCs; sphingomyelins, SMs; cholesterol esters, CEs; diacylglycerols, DAGs; and triacylglycerols, TAGs) were profiled using 10 μL of plasma separated by reversed phase chromatography after isopropanol extraction [2]. Nomenclature for lipid analytes entailed an initial number, denoting the total number of carbons in the lipid acyl chain, and a second number following the colon, denoting the total number of double bonds. For negatively charged polar metabolites (organic acids, sugars, and bile acids), 30 μL of plasma were extracted using four volumes of methanol and water following by chromatography using aLuna NH_2_ column (Phenomenex). Mass spectrometry data were acquired using a 4000 or 5500 QTRAP triple quadrupole mass spectrometer (AB SCIEX) using ESI, multiple reaction monitoring, and Q1 scans. In order to standardize measurements, a pooled plasma sample was run for every 20 participant samples, and experimental data were indexed to the nearest pooled plasma.

**Table A. Metabolites assayed and descriptions**

| **Method** |  |  |  |
| --- | --- | --- | --- |
|  |  |  |  |
| **Polar, positive charge** | 3-hydroxyanthranilic acid | creatine | ornithine |
|  | 5-hydroxyindoleacetic acid | creatinine | phenylalanine |
|  | alanine | dimethylglycine | proline |
|  | allantoin | gamma-aminoisobutyric acid | S-adenosylhomocysteine |
|  | aminoisobutyric acid | glutamate | serine |
|  | anthranilic acid | glutamine | serotonin |
|  | arginine | glycero-phospho-choline | symmetric dimethyarginine |
|  | argininosuccinate | glycerol | taurine |
|  | asparagine | glycine | thiamine |
|  | aspartate | histidine | threonine |
|  | asymmetric dimethylarginine | hydroxyproline | thyroxine |
|  | betaine | isoleucine | triiodothyronine |
|  | carbamoylalanine | kynurenic acid | trimethylamine |
|  | carnitine | leucine | tryptophan |
|  | carnosine | lysine | tyrosine |
|  | choline | methionine | valine |
|  | citrulline | NG-monomethyl-arginine | xanthosine |
|  | cotinine | niacinamide |  |
|  |  |  |  |
| **Polar, negative charge** | 2-hydroxyglutaric acid | glucuronate | pantothenic acid |
|  | 3-hydroxyphenylacetic acid | glycerol-3-phosphate | phosphocreatine |
|  | 3-methyladipic acid; pimelic acid | glycocholate | phosphoenolpyruvic acid |
|  | aconitate | glycodeoxycholate; glycochenodeoxycholate | propionic acid |
|  | adenosine | guanosine diphosphate | pyridoxate |
|  | adenosine diphosphate | guanosine monophosphate | pyruvate |
|  | adenosine monophosphate | hippuric acid | quinolinic acid |
|  | adipic acid | hyodeoxycholate; ursodeoxycholate; chenodeoxycholate; deoxycholate | ribose-5-phosphate; ribulose-5-phosphate |
|  | alpha-glycerophosphate | hypoxanthine | salicylurate |
|  | alpha-hydroxybutyric acid | indole-3-propionate | sorbitol |
|  | alpha-ketoglutarate | indoxylsulfate | suberic acid |
|  | aminoadipic acid | inosine | sucrose |
|  | beta-hydroxybutyric acid | inositol | taurocholate |
|  | Citrate | isocitrate | taurodeoxycholate; taurochenodeoxycholate |
|  | cyclic adenosine monophosphate | kynurenine | uric acid |
|  | cystathionine | Lactate | uridine |
|  | fructose-1-phosphate; fructose-6-phosphate; glucose-1-phosphate; glucose-6-phosphate | lactose | uridine diphosphate |
|  | fructose; glucose; galactose | Malate | uridine diphosphate glucose; uridine diphosphate galactose |
|  | fumarate; maleate; alpha-ketoisovalerate | orotic acid | xanthine |
|  | gentisic acid | oxalate | xanthurenate |
|  |  |  |  |
| **Lipid** | CE 14:0 | PC 32:0 | TAG 44:1 |
|  | CE 16:0 | PC 32:1 | TAG 46:0 |
|  | CE 16:1 | PC 32:2 | TAG 46:1 |
|  | CE 18:0 | PC 34:1 | TAG 46:2 |
|  | CE 18:1 | PC 34:2 | TAG 48:0 |
|  | CE 18:2 | PC 34:3 | TAG 48:1 |
|  | CE 18:3 | PC 34:4 | TAG 48:2 |
|  | CE 20:3 | PC 36:1 | TAG 48:3 |
|  | CE 20:4 | PC 36:2 | TAG 48:4 |
|  | CE 20:5 | PC 36:3 | TAG 50:1 |
|  | CE 22:6 | PC 38:2 | TAG 50:2 |
|  | DAG 34:1 | PC 38:3 | TAG 50:3 |
|  | DAG 34:2 | PC 38:4 | TAG 50:4 |
|  | DAG 36:1 | PC 38:5 | TAG 50:5 |
|  | DAG 36:2 | PC 38:6 | TAG 52:1 |
|  | LPC 14:0 | PC 40:6 | TAG 52:2 |
|  | LPC 16:0 | PCA 36:4 | TAG 52:3 |
|  | LPC 16:1 | PCB 36:4 | TAG 52:4 |
|  | LPC 18:0 | SM 14:0 | TAG 52:5 |
|  | LPC 18:1 | SM 16:0 | TAG 52:6 |
|  | LPC 18:2 | SM 16:1 | TAG 54:1 |
|  | LPC 20:3 | SM 18:0 | TAG 54:2 |
|  | LPC 20:4 | SM 18:1 | TAG 54:3 |
|  | LPC 20:5 | SM 22:0 | TAG 54:4 |
|  | LPC 22:6 | SM 22:1 | TAG 54:5 |
|  | LPE 16:0 | SM 24:0 | TAG 54:6 |
|  | LPE 18:0 | SM 24:1 | TAG 54:7 |
|  | LPE 18:1 |  | TAG 54:8 |
|  | LPE 18:2 |  | TAG 54:9 |
|  | LPE 20:4 |  | TAG 56:10 |
|  | LPE 22:6 |  | TAG 56:2 |
|  |  |  | TAG 56:3 |
|  |  |  | TAG 56:4 |
|  |  |  | TAG 56:5 |
|  |  |  | TAG 56:6 |
|  |  |  | TAG 56:7 |
|  |  |  | TAG 56:8 |
|  |  |  | TAG 56:9 |
|  |  |  | TAG 58:10 |
|  |  |  | TAG 58:11 |
|  |  |  | TAG 58:12 |
|  |  |  | TAG 58:6 |
|  |  |  | TAG 58:7 |
|  |  |  | TAG 58:8 |
|  |  |  | TAG 58:9 |
|  |  |  | TAG 60:12 |

**Table B**. Correlation of metabolic traits across examinations

| **Trait** |  | **Exam 5** | **Exam 6** | **Exam 7** | **Exam 8** |
| --- | --- | --- | --- | --- | --- |
|  |  | (1991-1995) | (1995-1998) | (1998-2001) | (2005-2008) |
|  |  |  |  |  |  |
| BMI, kg/m^2^ | Mean (SD) | 27.5 (4.9) | 28.0 (5.2) | 28.3 (5.4) | 28.3 (5.4) |
|  | Exam 5 (r) |  | 0.93 | 0.89 | 0.83 |
|  | Exam 6 (r) |  |  | 0.94 | 0.88 |
|  | Exam 7 (r) |  |  |  | 0.9 |
|  |  |  |  |  |  |
| Waist circumference (cm) | Mean (SD) | 93 (14) | 98 (14) | 100 (14) | 102 (14) |
|  | Exam 5 (r) |  | 0.85 | 0.81 | 0.74 |
|  | Exam 6 (r) |  |  | 0.92 | 0.86 |
|  | Exam 7 (r) |  |  |  | 0.88 |
|  |  |  |  |  |  |
| Fasting glucose (mg/dl) | Mean (SD) | 101 (27) | 104 (27) | 105 (26) | 107 (24) |
|  | Exam 5 (r) |  | 0.70 | 0.57 | 0.45 |
|  | Exam 6 (r) |  |  | 0.76 | 0.66 |
|  | Exam 7 (r) |  |  |  | 0.68 |
|  |  |  |  |  |  |
| HDL cholesterol (mg/dl) | Mean (SD) | 50 (15) | 51 (16) | 53 (17) | 57 (18) |
|  | Exam 5 (r) |  | 0.83 | 0.81 | 0.77 |
|  | Exam 6 (r) |  |  | 0.84 | 0.79 |
|  | Exam 7 (r) |  |  |  | 0.81 |
|  |  |  |  |  |  |
| log-triglycerides | Mean (SD) | 4.8 (0.6) | 4.8 (0.5) | 4.8 (0.5) | 4.7 (0.5) |
|  | Exam 5 (r) |  | 0.72 | 0.65 | 0.56 |
|  | Exam 6 (r) |  |  | 0.72 | 0.6 |
|  | Exam 7 (r) |  |  |  | 0.63 |
|  |  |  |  |  |  |
| Systolic BP, mmHg | Mean (SD) | 128 (20) | 132 (21) | 131 (20) | 133 (19) |
|  | Exam 5 (r) |  | 0.69 | 0.63 | 0.51 |
|  | Exam 6 (r) |  |  | 0.71 | 0.56 |
|  | Exam 7 (r) |  |  |  | 0.57 |
|  |  |  |  |  |  |
| Diastolic BP, mmHg | Mean (SD) | 76 (11) | 77 (10) | 76 (10) | 76 (10) |
|  | Exam 5 (r) |  | 0.61 | 0.53 | 0.36 |
|  | Exam 6 (r) |  |  | 0.63 | 0.46 |
|  | Exam 7 (r) |  |  |  | 0.51 |

Data are presented as mean(SD) for each exam and estimated Pearson correlation coefficient between each pair of exams.

**Table C.** Association of non-lipid metabolites and HOMA-IR in obese and non-obese individuals

|  | **Non-obese (n=1680)** | | | **Obese (n=511)** | | | **Interaction** |
| --- | --- | --- | --- | --- | --- | --- | --- |
| **Metabolite** | **beta** | **s.e.** | **P-value** | **beta** | **s.e.** | **P-value** | **P-value** |
| 2-hydroxyglutaric acid | -0.01 | 0.02 | 0.58 | 0.05 | 0.03 | 0.13 | 0.19 |
| 3-hydroxyanthranilic acid | 0.04 | 0.02 | 0.11 | 0.04 | 0.04 | 0.22 | 0.82 |
| 3-hydroxyphenylacetic acid | 0.01 | 0.02 | 0.52 | 0.12 | 0.03 | 3.47E-04 | 1.40E-03 |
| 3-methyladipic acid; pimelic acid | 0.01 | 0.02 | 0.49 | 0.02 | 0.04 | 0.65 | 0.53 |
| 5-hydroxyindoleacetic acid | -0.01 | 0.02 | 0.57 | 0.04 | 0.04 | 0.21 | 0.07 |
| aconitate | 0.04 | 0.02 | 0.04 | 0.11 | 0.04 | 2.88E-03 | 0.48 |
| adenosine | 0.00 | 0.02 | 0.85 | 0.03 | 0.03 | 0.43 | 0.71 |
| adenosine diphosphate | -0.04 | 0.02 | 0.03 | 0.03 | 0.03 | 0.42 | 0.13 |
| adenosine monophosphate | 0.09 | 0.02 | 1.64E-06 | 0.05 | 0.03 | 0.18 | 0.16 |
| adipic acid | 0.03 | 0.02 | 0.09 | 0.02 | 0.03 | 0.55 | 0.55 |
| alanine | 0.08 | 0.02 | 6.46E-05 | 0.11 | 0.04 | 2.40E-03 | 0.30 |
| allantoin | 0.02 | 0.02 | 0.20 | 0.02 | 0.03 | 0.61 | 0.20 |
| alpha-glycerophosphate | -0.05 | 0.02 | 0.01 | -0.04 | 0.03 | 0.24 | 0.50 |
| alpha-hydroxybutyric acid | 0.01 | 0.02 | 0.63 | 0.05 | 0.04 | 0.16 | 0.38 |
| alpha-ketoglutarate | 0.06 | 0.02 | 0.01 | 0.13 | 0.03 | 9.65E-05 | 0.05 |
| aminoadipic acid | 0.15 | 0.02 | 5.57E-11 | 0.13 | 0.04 | 5.15E-04 | 0.25 |
| aminoisobutyric acid | -0.04 | 0.02 | 0.02 | -0.04 | 0.03 | 0.19 | 0.65 |
| anthranilic acid | -0.01 | 0.02 | 0.78 | 0.03 | 0.03 | 0.44 | 0.42 |
| arginine | 0.01 | 0.02 | 0.55 | -0.02 | 0.03 | 0.53 | 0.68 |
| argininosuccinate | 0.03 | 0.03 | 0.43 | 0.02 | 0.05 | 0.70 | 0.68 |
| asparagine | -0.12 | 0.02 | 8.48E-11 | -0.12 | 0.03 | 2.36E-04 | 0.51 |
| aspartate | -0.04 | 0.03 | 0.19 | -0.01 | 0.04 | 0.86 | 0.23 |
| asymmetric dimethylarginine | 0.00 | 0.02 | 0.79 | 0.07 | 0.03 | 0.05 | 0.03 |
| beta-hydroxybutyric acid | -0.02 | 0.02 | 0.25 | -0.01 | 0.04 | 0.89 | 0.84 |
| betaine | -0.04 | 0.02 | 0.02 | -0.03 | 0.04 | 0.41 | 0.11 |
| carbamoylalanine | 0.08 | 0.02 | 2.02E-05 | 0.10 | 0.03 | 1.62E-03 | 0.21 |
| carnitine | -0.02 | 0.02 | 0.36 | 0.07 | 0.03 | 0.05 | 1.70E-03 |
| carnosine | -0.03 | 0.03 | 0.32 | 0.02 | 0.04 | 0.57 | 0.29 |
| choline | -0.04 | 0.02 | 0.06 | -0.03 | 0.03 | 0.29 | 0.21 |
| citrate | 0.02 | 0.02 | 0.37 | 0.02 | 0.04 | 0.54 | 0.49 |
| citrulline | -0.03 | 0.02 | 0.12 | -0.03 | 0.03 | 0.44 | 0.24 |
| cotinine | -0.03 | 0.02 | 0.14 | -0.02 | 0.03 | 0.50 | 0.41 |
| creatine | 0.03 | 0.02 | 0.21 | 0.00 | 0.04 | 0.96 | 0.01 |
| creatinine | 0.03 | 0.02 | 0.24 | 0.01 | 0.03 | 0.70 | 0.08 |
| cyclic adenosine monophosphate | -0.04 | 0.02 | 0.08 | 0.03 | 0.04 | 0.34 | 0.06 |
| cystathionine | 0.09 | 0.02 | 3.12E-05 | 0.13 | 0.03 | 4.58E-05 | 0.16 |
| dimethylglycine | 0.04 | 0.02 | 0.02 | 0.09 | 0.03 | 0.01 | 0.04 |
| fructose-1-phosphate; fructose-6-phosphate; glucose-1-phosphate; glucose-6-phosphate | 0.07 | 0.02 | 1.10E-03 | 0.08 | 0.04 | 0.03 | 0.94 |
| fructose; glucose; galactose | 0.15 | 0.03 | 1.06E-07 | 0.20 | 0.05 | 5.31E-05 | 0.62 |
| fumarate; maleate; alpha-ketoisovalerate | -0.02 | 0.02 | 0.29 | 0.05 | 0.03 | 0.12 | 0.11 |
| gamma-aminoisobutyric acid | -0.04 | 0.02 | 0.05 | 0.02 | 0.04 | 0.69 | 0.24 |
| gentisic acid | -0.02 | 0.02 | 0.30 | 0.01 | 0.03 | 0.83 | 0.34 |
| glucuronate | -0.02 | 0.02 | 0.44 | 0.05 | 0.03 | 0.15 | 0.05 |
| glutamate | -0.01 | 0.02 | 0.69 | 0.03 | 0.03 | 0.32 | 0.03 |
| glutamine | -0.02 | 0.02 | 0.32 | -0.08 | 0.03 | 0.01 | 0.06 |
| glycero-phospho-choline | 0.04 | 0.02 | 0.01 | -0.04 | 0.03 | 0.25 | 0.35 |
| glycerol | 0.01 | 0.02 | 0.56 | 0.10 | 0.04 | 0.02 | 0.27 |
| glycerol-3-phosphate | 0.03 | 0.02 | 0.09 | 0.03 | 0.04 | 0.45 | 0.71 |
| glycine | -0.03 | 0.02 | 0.10 | -0.10 | 0.03 | 1.82E-03 | 0.03 |
| glycocholate | 0.03 | 0.02 | 0.09 | 0.12 | 0.03 | 4.50E-04 | 3.89E-03 |
| glycodeoxycholate; glycochenodeoxycholate | 0.06 | 0.02 | 2.41E-03 | 0.13 | 0.03 | 3.38E-05 | 0.04 |
| guanosine diphosphate | 0.03 | 0.02 | 0.16 | 0.05 | 0.04 | 0.18 | 0.74 |
| guanosine monophosphate | 0.05 | 0.02 | 0.01 | 0.03 | 0.04 | 0.38 | 0.57 |
| hippuric acid | -0.01 | 0.02 | 0.51 | 0.01 | 0.03 | 0.82 | 0.80 |
| histidine | -0.02 | 0.02 | 0.22 | -0.05 | 0.03 | 0.12 | 1.00 |
| hydroxyproline | 0.02 | 0.02 | 0.33 | 0.05 | 0.03 | 0.09 | 0.08 |
| hyodeoxycholate; ursodeoxycholate; chenodeoxycholate; deoxycholate | 0.05 | 0.02 | 0.01 | 0.10 | 0.04 | 4.65E-03 | 0.35 |
| hypoxanthine | -0.03 | 0.02 | 0.12 | -0.01 | 0.03 | 0.67 | 0.83 |
| indole-3-propionate | -0.02 | 0.02 | 0.29 | 0.05 | 0.04 | 0.20 | 0.06 |
| indoxylsulfate | 0.08 | 0.02 | 2.44E-04 | 0.04 | 0.03 | 0.19 | 0.46 |
| inosine | 0.00 | 0.02 | 0.82 | 0.11 | 0.04 | 3.37E-03 | 0.01 |
| inositol | -0.01 | 0.02 | 0.69 | 0.09 | 0.04 | 0.01 | 0.02 |
| isocitrate | 0.11 | 0.02 | 3.95E-06 | 0.15 | 0.04 | 2.21E-05 | 0.62 |
| isoleucine | 0.08 | 0.02 | 8.90E-04 | 0.10 | 0.04 | 0.01 | 0.01 |
| kynurenic acid | 0.04 | 0.02 | 0.05 | 0.01 | 0.03 | 0.65 | 0.90 |
| kynurenine | 0.06 | 0.02 | 0.01 | 0.14 | 0.03 | 2.93E-05 | 0.02 |
| lactate | 0.14 | 0.02 | 1.09E-09 | 0.19 | 0.03 | 4.05E-08 | 0.11 |
| lactose | 0.03 | 0.02 | 0.08 | 0.11 | 0.04 | 2.24E-03 | 0.34 |
| leucine | 0.05 | 0.02 | 0.02 | 0.07 | 0.04 | 0.10 | 0.03 |
| lysine | -0.02 | 0.02 | 0.27 | -0.03 | 0.03 | 0.41 | 0.39 |
| malate | 0.01 | 0.02 | 0.60 | 0.04 | 0.03 | 0.18 | 0.24 |
| methionine | 0.03 | 0.02 | 0.17 | 0.01 | 0.03 | 0.71 | 0.14 |
| NG-monomethyl-arginine | -0.05 | 0.02 | 0.01 | 0.02 | 0.03 | 0.59 | 0.02 |
| niacinamide | 0.02 | 0.02 | 0.30 | 0.04 | 0.04 | 0.26 | 0.26 |
| ornithine | 0.04 | 0.02 | 0.04 | 0.01 | 0.03 | 0.67 | 0.35 |
| orotic acid | -0.02 | 0.02 | 0.27 | 0.08 | 0.04 | 0.03 | 0.02 |
| oxalate | -0.04 | 0.02 | 0.05 | -0.07 | 0.04 | 0.10 | 0.46 |
| pantothenic acid | -0.02 | 0.02 | 0.29 | -0.01 | 0.04 | 0.72 | 0.94 |
| phenylalanine | 0.05 | 0.02 | 0.02 | 0.09 | 0.04 | 0.01 | 0.07 |
| phosphocreatine | -0.04 | 0.02 | 0.06 | -0.07 | 0.03 | 0.04 | 0.32 |
| phosphoenolpyruvic acid | -0.02 | 0.02 | 0.40 | 0.03 | 0.04 | 0.50 | 0.20 |
| proline | 0.07 | 0.02 | 2.80E-04 | 0.10 | 0.04 | 4.97E-03 | 0.02 |
| propionic acid | 0.04 | 0.02 | 0.05 | 0.06 | 0.04 | 0.10 | 0.40 |
| pyridoxate | -0.05 | 0.02 | 0.02 | -0.03 | 0.03 | 0.44 | 0.81 |
| pyruvate | 0.04 | 0.02 | 0.05 | 0.02 | 0.04 | 0.60 | 0.61 |
| quinolinic acid | 0.10 | 0.02 | 9.45E-06 | 0.11 | 0.03 | 9.07E-04 | 0.94 |
| ribose-5-phosphate; ribulose-5-phosphate | 0.04 | 0.02 | 0.04 | 0.06 | 0.04 | 0.10 | 0.57 |
| S-adenosylhomocysteine | 0.07 | 0.03 | 0.04 | 0.02 | 0.04 | 0.56 | 0.63 |
| salicylurate | -0.01 | 0.02 | 0.49 | 0.05 | 0.03 | 0.10 | 0.03 |
| serine | -0.02 | 0.02 | 0.20 | -0.08 | 0.03 | 0.01 | 0.07 |
| serotonin | 0.00 | 0.02 | 0.97 | 0.02 | 0.03 | 0.62 | 0.90 |
| sorbitol | 0.02 | 0.02 | 0.25 | 0.07 | 0.03 | 0.05 | 0.31 |
| suberic acid | 0.04 | 0.02 | 0.10 | 0.06 | 0.05 | 0.21 | 0.75 |
| sucrose | 0.02 | 0.02 | 0.23 | 0.04 | 0.03 | 0.14 | 0.47 |
| symmetric dimethyarginine | -0.06 | 0.02 | 0.01 | -0.05 | 0.03 | 0.09 | 0.40 |
| taurine | -0.01 | 0.02 | 0.62 | -0.02 | 0.03 | 0.50 | 0.48 |
| taurocholate | 0.09 | 0.02 | 1.45E-05 | 0.18 | 0.03 | 1.11E-08 | 0.01 |
| taurodeoxycholate; taurochenodeoxycholate | 0.05 | 0.03 | 0.04 | 0.13 | 0.03 | 8.10E-05 | 0.02 |
| thiamine | 0.00 | 0.02 | 0.81 | 0.00 | 0.03 | 0.94 | 0.42 |
| threonine | -0.01 | 0.02 | 0.49 | -0.04 | 0.03 | 0.21 | 0.98 |
| thyroxine | 0.05 | 0.02 | 0.01 | -0.02 | 0.03 | 0.61 | 0.06 |
| triiodothyronine | 0.07 | 0.02 | 3.67E-03 | 0.08 | 0.04 | 0.06 | 0.82 |
| trimethylamine | 0.01 | 0.02 | 0.42 | 0.00 | 0.03 | 0.90 | 0.55 |
| tryptophan | 0.02 | 0.02 | 0.33 | 0.06 | 0.03 | 0.07 | 0.01 |
| tyrosine | 0.05 | 0.02 | 0.02 | 0.11 | 0.03 | 1.52E-03 | 0.01 |
| uric acid | 0.03 | 0.02 | 0.25 | 0.04 | 0.03 | 0.20 | 0.30 |
| uridine | -0.06 | 0.02 | 2.64E-03 | -0.08 | 0.04 | 0.03 | 0.95 |
| uridine diphosphate | 0.06 | 0.02 | 0.02 | 0.09 | 0.05 | 0.05 | 0.71 |
| uridine diphosphate glucose; uridine diphosphate galactose | 0.02 | 0.02 | 0.34 | 0.09 | 0.04 | 0.02 | 0.24 |
| valine | 0.08 | 0.02 | 2.21E-04 | 0.11 | 0.04 | 3.51E-03 | 0.06 |
| xanthine | 0.05 | 0.02 | 0.02 | 0.02 | 0.03 | 0.54 | 0.75 |
| xanthosine | 0.04 | 0.02 | 0.06 | 0.10 | 0.03 | 2.73E-03 | 0.08 |
| xanthurenate | 0.01 | 0.02 | 0.63 | -0.01 | 0.04 | 0.84 | 0.65 |

Red font indicates significant association using Bonferroni-corrected P-value threshold of P<0.00023, and blue font a suggestive P-value of P<0.001.

**Table D.** Association of lipid metabolites and HOMA-IR in obese and non-obese individuals

|  | **Non-obese (n=1680)** | | | **Obese (n=511)** | | | **Interaction** |
| --- | --- | --- | --- | --- | --- | --- | --- |
| **Metabolite** | **beta** | **s.e.** | **P-value** | **beta** | **s.e.** | **P-value** | **P-value** |
| CE 14:0 | -0.03 | 0.02 | 0.17 | 0.03 | 0.03 | 0.38 | 0.15 |
| CE 16:0 | -0.04 | 0.02 | 0.12 | -0.02 | 0.03 | 0.52 | 0.83 |
| CE 16:1 | -0.03 | 0.02 | 0.12 | 0.03 | 0.03 | 0.44 | 0.76 |
| CE 18:0 | -0.02 | 0.02 | 0.27 | 0.04 | 0.03 | 0.21 | 0.52 |
| CE 18:1 | -0.08 | 0.02 | 1.18E-03 | 0.00 | 0.04 | 0.97 | 0.63 |
| CE 18:2 | -0.03 | 0.03 | 0.28 | 0.02 | 0.04 | 0.60 | 0.94 |
| CE 18:3 | -0.01 | 0.02 | 0.59 | -0.01 | 0.04 | 0.79 | 0.56 |
| CE 20:3 | 0.03 | 0.02 | 0.27 | 0.06 | 0.04 | 0.10 | 0.26 |
| CE 20:4 | 0.00 | 0.03 | 0.95 | -0.01 | 0.04 | 0.90 | 0.27 |
| CE 20:5 | -0.02 | 0.02 | 0.30 | -0.04 | 0.04 | 0.32 | 0.44 |
| CE 22:6 | 0.00 | 0.03 | 0.98 | 0.04 | 0.04 | 0.25 | 0.67 |
| DAG 34:1 | 0.12 | 0.04 | 6.39E-04 | 0.15 | 0.06 | 0.02 | 0.28 |
| DAG 34:2 | 0.15 | 0.04 | 8.82E-05 | 0.14 | 0.06 | 0.03 | 0.51 |
| DAG 36:1 | 0.14 | 0.04 | 9.24E-05 | 0.09 | 0.06 | 0.12 | 0.92 |
| DAG 36:2 | 0.05 | 0.04 | 0.27 | -0.09 | 0.07 | 0.22 | 0.92 |
| LPC 14:0 | 0.04 | 0.02 | 0.07 | -0.05 | 0.04 | 0.15 | 0.18 |
| LPC 16:0 | -0.01 | 0.02 | 0.69 | -0.05 | 0.04 | 0.14 | 0.93 |
| LPC 16:1 | -0.06 | 0.02 | 0.01 | -0.06 | 0.04 | 0.13 | 0.75 |
| LPC 18:0 | 0.03 | 0.02 | 0.24 | -0.04 | 0.04 | 0.32 | 0.60 |
| LPC 18:1 | -0.05 | 0.02 | 0.02 | -0.12 | 0.03 | 4.84E-04 | 0.50 |
| LPC 18:2 | -0.06 | 0.02 | 4.36E-03 | -0.11 | 0.04 | 2.83E-03 | 0.61 |
| LPC 20:3 | -0.02 | 0.02 | 0.38 | -0.05 | 0.03 | 0.13 | 0.86 |
| LPC 20:4 | -0.04 | 0.02 | 0.08 | -0.13 | 0.03 | 5.79E-05 | 0.17 |
| LPC 20:5 | -0.01 | 0.02 | 0.49 | -0.15 | 0.03 | 1.16E-05 | 0.04 |
| LPC 22:6 | -0.03 | 0.02 | 0.12 | -0.10 | 0.04 | 0.01 | 0.67 |
| LPE16:0 | -0.09 | 0.02 | 1.62E-04 | -0.05 | 0.04 | 0.20 | 0.29 |
| LPE18:0 | -0.04 | 0.02 | 0.09 | -0.08 | 0.03 | 0.01 | 0.39 |
| LPE18:1 | -0.04 | 0.02 | 0.07 | -0.04 | 0.03 | 0.19 | 0.52 |
| LPE18:2 | -0.07 | 0.02 | 1.42E-03 | -0.04 | 0.03 | 0.28 | 0.16 |
| LPE20:4 | -0.08 | 0.02 | 6.90E-05 | -0.10 | 0.03 | 2.11E-03 | 0.92 |
| LPE22:6 | 0.02 | 0.02 | 0.38 | -0.03 | 0.04 | 0.46 | 0.80 |
| PC 32:0 | -0.04 | 0.02 | 0.07 | 0.01 | 0.03 | 0.73 | 0.16 |
| PC 32:1 | -0.05 | 0.02 | 0.02 | 0.02 | 0.04 | 0.69 | 0.21 |
| PC 32:2 | 0.01 | 0.02 | 0.66 | -0.01 | 0.04 | 0.76 | 0.42 |
| PC 34:1 | -0.08 | 0.02 | 1.91E-04 | -0.07 | 0.04 | 0.04 | 0.81 |
| PC 34:2 | 0.00 | 0.02 | 0.88 | -0.02 | 0.03 | 0.49 | 0.60 |
| PC 34:3 | -0.08 | 0.02 | 5.88E-04 | -0.07 | 0.04 | 0.09 | 0.69 |
| PC 34:4 | 0.00 | 0.02 | 0.86 | -0.05 | 0.04 | 0.15 | 0.11 |
| PC 36:1 | 0.00 | 0.02 | 0.99 | 0.04 | 0.04 | 0.27 | 0.27 |
| PC 36:2 | -0.03 | 0.02 | 0.19 | 0.03 | 0.04 | 0.45 | 0.16 |
| PC 36:3 | -0.07 | 0.02 | 2.96E-03 | -0.06 | 0.04 | 0.08 | 0.74 |
| PC 38:2 | -0.05 | 0.02 | 0.01 | 0.00 | 0.04 | 0.95 | 0.47 |
| PC 38:3 | -0.03 | 0.02 | 0.24 | 0.03 | 0.04 | 0.38 | 0.32 |
| PC 38:4 | -0.02 | 0.02 | 0.38 | -0.03 | 0.03 | 0.42 | 0.46 |
| PC 38:5 | -0.06 | 0.02 | 0.01 | -0.10 | 0.03 | 0.01 | 0.27 |
| PC 38:6 | -0.05 | 0.02 | 0.02 | -0.06 | 0.04 | 0.08 | 0.79 |
| PC 40:6 | 0.01 | 0.02 | 0.62 | 0.04 | 0.04 | 0.27 | 0.46 |
| PCA 36:4 | -0.04 | 0.02 | 0.04 | -0.07 | 0.03 | 0.04 | 0.61 |
| PCB 36:4 | -0.03 | 0.02 | 0.22 | -0.09 | 0.03 | 0.01 | 0.05 |
| SM 14:0 | 0.01 | 0.02 | 0.80 | -0.04 | 0.03 | 0.21 | 0.24 |
| SM 16:0 | 0.00 | 0.02 | 0.90 | -0.02 | 0.04 | 0.67 | 0.56 |
| SM 16:1 | -0.04 | 0.02 | 0.10 | -0.06 | 0.03 | 0.07 | 0.06 |
| SM 18:0 | 0.04 | 0.02 | 0.05 | 0.05 | 0.03 | 0.10 | 0.53 |
| SM 18:1 | 0.01 | 0.02 | 0.56 | 0.00 | 0.04 | 1.00 | 0.03 |
| SM 22:0 | 0.01 | 0.02 | 0.64 | -0.02 | 0.03 | 0.62 | 0.55 |
| SM 22:1 | -0.02 | 0.02 | 0.28 | -0.08 | 0.04 | 0.04 | 0.15 |
| SM 24:0 | -0.04 | 0.02 | 0.08 | -0.04 | 0.04 | 0.26 | 0.23 |
| SM 24:1 | -0.03 | 0.02 | 0.18 | 0.01 | 0.03 | 0.87 | 0.55 |
| TAG 44:1 | 0.11 | 0.02 | 4.07E-06 | 0.12 | 0.04 | 4.88E-03 | 0.33 |
| TAG 46:0 | 0.08 | 0.03 | 2.30E-03 | 0.18 | 0.05 | 2.07E-04 | 0.02 |
| TAG 46:1 | 0.10 | 0.03 | 1.25E-04 | 0.16 | 0.05 | 7.49E-04 | 0.14 |
| TAG 46:2 | 0.11 | 0.03 | 1.63E-04 | 0.16 | 0.05 | 1.20E-03 | 0.17 |
| TAG 48:0 | 0.09 | 0.02 | 3.30E-04 | 0.18 | 0.04 | 5.49E-06 | 0.02 |
| TAG 48:1 | 0.08 | 0.03 | 2.14E-03 | 0.17 | 0.05 | 2.21E-04 | 0.05 |
| TAG 48:2 | 0.09 | 0.03 | 0.01 | 0.17 | 0.06 | 2.06E-03 | 0.14 |
| TAG 48:3 | 0.11 | 0.03 | 8.45E-04 | 0.13 | 0.06 | 0.03 | 0.43 |
| TAG 48:4 | 0.08 | 0.03 | 2.32E-03 | 0.03 | 0.04 | 0.40 | 0.94 |
| TAG 50:1 | 0.08 | 0.03 | 3.06E-03 | 0.21 | 0.06 | 5.26E-04 | 0.03 |
| TAG 50:2 | 0.04 | 0.03 | 0.18 | 0.16 | 0.05 | 1.45E-03 | 0.08 |
| TAG 50:3 | 0.03 | 0.04 | 0.52 | 0.21 | 0.07 | 1.96E-03 | 0.11 |
| TAG 50:4 | 0.07 | 0.04 | 0.08 | 0.13 | 0.07 | 0.06 | 0.36 |
| TAG 50:5 | 0.05 | 0.03 | 0.05 | 0.03 | 0.05 | 0.47 | 0.85 |
| TAG 52:1 | 0.13 | 0.03 | 9.66E-05 | 0.28 | 0.06 | 3.18E-06 | 0.04 |
| TAG 52:2 | 0.02 | 0.03 | 0.57 | 0.14 | 0.05 | 0.01 | 0.12 |
| TAG 52:3 | 0.01 | 0.03 | 0.66 | 0.00 | 0.05 | 0.92 | 0.99 |
| TAG 52:4 | 0.00 | 0.03 | 0.87 | 0.01 | 0.05 | 0.88 | 0.78 |
| TAG 52:5 | 0.02 | 0.03 | 0.60 | 0.01 | 0.05 | 0.85 | 0.77 |
| TAG 52:6 | 0.04 | 0.03 | 0.17 | 0.02 | 0.05 | 0.66 | 0.98 |
| TAG 54:1 | 0.15 | 0.03 | 3.44E-06 | 0.26 | 0.07 | 1.27E-04 | 0.02 |
| TAG 54:2 | 0.08 | 0.04 | 0.02 | 0.15 | 0.06 | 0.01 | 0.24 |
| TAG 54:3 | 0.00 | 0.03 | 0.88 | 0.02 | 0.05 | 0.67 | 0.44 |
| TAG 54:4 | -0.03 | 0.02 | 0.22 | -0.03 | 0.04 | 0.37 | 0.75 |
| TAG 54:5 | -0.01 | 0.02 | 0.62 | -0.05 | 0.04 | 0.17 | 0.71 |
| TAG 54:6 | 0.01 | 0.03 | 0.82 | -0.01 | 0.04 | 0.80 | 0.84 |
| TAG 54:7 | 0.02 | 0.03 | 0.45 | -0.02 | 0.04 | 0.71 | 0.94 |
| TAG 54:8 | 0.05 | 0.03 | 0.11 | -0.01 | 0.05 | 0.81 | 0.69 |
| TAG 54:9 | 0.01 | 0.02 | 0.60 | -0.02 | 0.04 | 0.64 | 0.93 |
| TAG 56:10 | -0.02 | 0.02 | 0.45 | -0.01 | 0.04 | 0.76 | 0.55 |
| TAG 56:2 | 0.08 | 0.03 | 0.02 | 0.14 | 0.08 | 0.07 | 0.09 |
| TAG 56:3 | -0.02 | 0.03 | 0.60 | -0.03 | 0.05 | 0.61 | 0.49 |
| TAG 56:4 | -0.05 | 0.03 | 0.07 | -0.05 | 0.04 | 0.21 | 0.58 |
| TAG 56:5 | -0.02 | 0.02 | 0.50 | -0.08 | 0.03 | 0.02 | 0.25 |
| TAG 56:6 | -0.01 | 0.02 | 0.71 | -0.05 | 0.03 | 0.15 | 0.49 |
| TAG 56:7 | 0.00 | 0.02 | 0.84 | -0.06 | 0.04 | 0.13 | 0.45 |
| TAG 56:8 | 0.00 | 0.02 | 0.88 | -0.02 | 0.04 | 0.59 | 0.93 |
| TAG 56:9 | 0.01 | 0.03 | 0.56 | -0.02 | 0.04 | 0.54 | 0.73 |
| TAG 58:10 | -0.01 | 0.02 | 0.78 | -0.03 | 0.04 | 0.47 | 1.00 |
| TAG 58:11 | -0.04 | 0.02 | 0.12 | -0.04 | 0.03 | 0.22 | 0.44 |
| TAG 58:12 | -0.06 | 0.02 | 0.01 | -0.01 | 0.04 | 0.71 | 0.53 |
| TAG 58:6 | 0.01 | 0.02 | 0.59 | 0.03 | 0.05 | 0.44 | 0.39 |
| TAG 58:7 | 0.03 | 0.02 | 0.17 | -0.01 | 0.04 | 0.75 | 0.72 |
| TAG 58:8 | 0.00 | 0.02 | 0.92 | -0.05 | 0.03 | 0.15 | 0.43 |
| TAG 58:9 | -0.01 | 0.02 | 0.75 | -0.03 | 0.04 | 0.41 | 0.91 |
| TAG 60:12 | -0.01 | 0.02 | 0.79 | 0.01 | 0.03 | 0.86 | 0.39 |

Red font indicates significant association using Bonferroni-corrected P-value threshold of P<0.00023, and blue font a suggestive P-value of P<0.001.

**References**

1. Wang TJ, Larson MG, Vasan RS, Cheng S, Rhee EP, et al. (2011) Metabolite profiles and the risk of developing diabetes. Nat Med 17: 448-453.

2. Rhee EP, Cheng S, Larson MG, Walford GA, Lewis GD, et al. Lipid profiling identifies a triacylglycerol signature of insulin resistance and improves diabetes prediction in humans. J Clin Invest 121: 1402-1411.
